# Macroscopic anatomy, radiography and computed tomography of normal paranasal sinuses of the adult one‐humped dromedary (*Camelus dromedarius*)

**DOI:** 10.1002/vms3.515

**Published:** 2021-05-01

**Authors:** Abdelmoneme Ben Khalifa, Ayoub Ben Braiek, Leila Belhaj Hmida, Walid Chandoul, Abdelhamid Mattoussi

**Affiliations:** ^1^ Department of Anatomy and Embryology Université de la Manouba Sidi Thabet Tunisia; ^2^ Ministry of Agriculture, Water Resources and Maritime Fisheries, Arrondissement de Production Animale de Médenine Médenine Tunisia

**Keywords:** anatomic sections, *Camelus dromedarius*, computed tomography, paranasal sinuses, radiography

## Abstract

In order to improve the current knowledge of the topography and the anatomy of the paranasal sinuses of the one‐humped dromedary (*Camelus dromedarius*), we applied both conventional and advanced imaging techniques, namely, radiography and computed tomography (CT). Twelve heads of healthy dromedaries were used; eight heads were dissected to obtain skulls for the fenestration of the sinuses, two heads underwent anatomical sections, and two heads were imaged respectively by radiography and CT. Sinus fenestration allowed observation of sinuses and their communications. In each dissected dromedary's head, the frontal sinus is a large compartment delimited by thick partitions. The sphenoid sinus is divided into small compartments by bony plates. The lacrimal sinus occupies a small cavity in the rostro‐medial lacrimal bone of the orbit. In all dissected heads, there was neither palatal sinus nor ventral conchal sinus. Five images obtained by CT were selected with an excellent correspondence with the anatomical sections. These images allowed a good differentiation between bones and sinus cavities. The visualisation of the sinus cavities and their anatomical limits has better quality using the CT compared to the radiography. Radiographic and CT images are therefore very useful for the interpretation of clinical imaging studies of the dromedary's paranasal sinuses.

## Graphical abstract

Our article is a complete atlas of anatomic, radiographic and tomodensitometric images of the paranasal sinuses of the dromedary. The location, topography and communications between the sinuses are well detailed

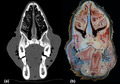

## 1 INTRODUCTION

The dromedary (*Camelus dromedarius*) population in North Africa was estimated to be 900,000, among them 231,000 in Tunisia (Sghaier, 2004). Dromedaries are present in arid and desert regions, they are kept under an extensive breeding system characterised by the mobility of herds between different regions of south Tunisia (Seddik et al., 2016). In the extreme environment conditions of Sahara, dromedaries are adapted to take advantage of the limited water and food resources to work and produce milk, meat and wool (Aljumaah et al., 2011). Dromedaries live in remote regions; veterinarians face difficulties in making diagnoses of several conditions, mainly because imaging tools are not available. For example, the exploration of the anatomical structures of the head and the evaluation of heads' soft tissues and oral and nasal cavities are still not well documented. Physical exploration and current diagnostic imaging techniques such as radiography and ultrasound provide limited information for the assessment of the health of a dromedary's head soft tissues (Byers et al., 2007).

Paranasal sinuses are air chambers carved into the thickness of the skull bones (Dyce et al., 2010). They have a complex spatial organisation that varies in mammals with their age. These structures enhance solidity of the skull without weighing it down.

Due to its availability, radiography is widely used for paranasal sinuses examination, but computed tomography (CT) has better performance and is nowadays the technique of choice (Langner, 2015). Indeed, CT allows performing thin slices, with a high spatial resolution of all superpositions of adjacent planes and a good resolution in contrast (Whyte et al., 2017). CT also allows a good anatomical analyses of both nasal and sinus cavities and the variations of pneumatisation that can modify the sinus reports leading to a significant advancement of diagnostic imaging in veterinary medicine.

The aim of the present study was to provide a comprehensive description of the normal anatomy of paranasal sinuses of the dromedary and its related structures by developing an atlas of radiographic, CT and macroscopic sections. This atlas will constitute an anatomical reference that could be used during clinical examination of dromedaries' paranasal sinuses.

## 2 MATERIALS AND METHODS

### 2.1 Animals

Twelve heads of adult healthy dromedaries (*Camelus dromedarius*), free of any disease affecting the skull, intended for human consumption and collected from the slaughterhouse of Medenine (southeast Tunisia), were used in the present study. All dromedary heads were severed at the atlanto‐occipital joint immediately after being slaughtered.

### 2.2 Preparation of dromedary's skeleton heads and bones' fenestrations

In order to obtain bone planes, eight dromedary heads were used. Muscles of both the superficial and deep plane and all head viscera (tongue, pharynx, glands etc.) were removed. The remaining soft tissue was eliminated by boiling the heads during approximately 6 hr. Finally, the heads were incubated in hydrogen peroxide at 35 volumes for 3 days to get clean and white bones. The dissection of sinuses was performed on the eight bony heads after fenestration of the frontal, nasal and maxillary bones of the head by tangential erosion with a disc wheel. Windows were made delicately by bone erosion in specific locations, depending on the studied sinus.

### 2.3 Dromedary's heads anatomical sections

Two frozen dromedary heads were used for the anatomical sections: one for the sagittal section and the second for cross‐sections. The latter was sliced transversely in series at approximately 1.5 cm intervals with a band saw. The sections were made from the interdental space just before the first premolar to the level of the third molar, corresponding to the level of the most caudal paranasal sinus, namely, the sphenoid sinus. Slices were numbered and delicately cleaned with tap water and light brushing. The caudal surface of each slice was immediately photographed. Cheek teeth and bony parts were used as landmarks to explore and describe the location and extension of structures and cavities.

### 2.4 Dromedary's head radiography

A digital radiography machine (ZooMax White DR Veterinary Radiographic System (CMP); parameters: 66 KVp, 10 mAs, 0,004 s and 100 cm FFD) was used for performing conventional lateral, dorsoventral and oblique radiographic views on a fresh dromedary head after being thoroughly cleaned.

### 2.5 Dromedary's head CT scan

One fresh dromedary head was thoroughly cleaned then examined by CT using the following parameters: 120 kV, 200 mA, slice thickness of 0.6 mm and spacing of 0.6 mm.

The head of the dromedary was placed in the scanner, and images were acquired. To assess the appearance of the nasal and oral cavities in CT scan, two windows were used by adjusting window widths (WW) and levels (WL) to bone (WW = 4,000; WL = 378) and soft tissues (WW = 372; WL = 58). The last was made for a better clarity of the associated soft tissues structures. We used RadiAnt DICOM Viewer to review the images.

## 3 RESULTS

### 3.1 Descriptive and topographic study

#### 3.1.1 Conchal sinus

**FIGURE 1:**
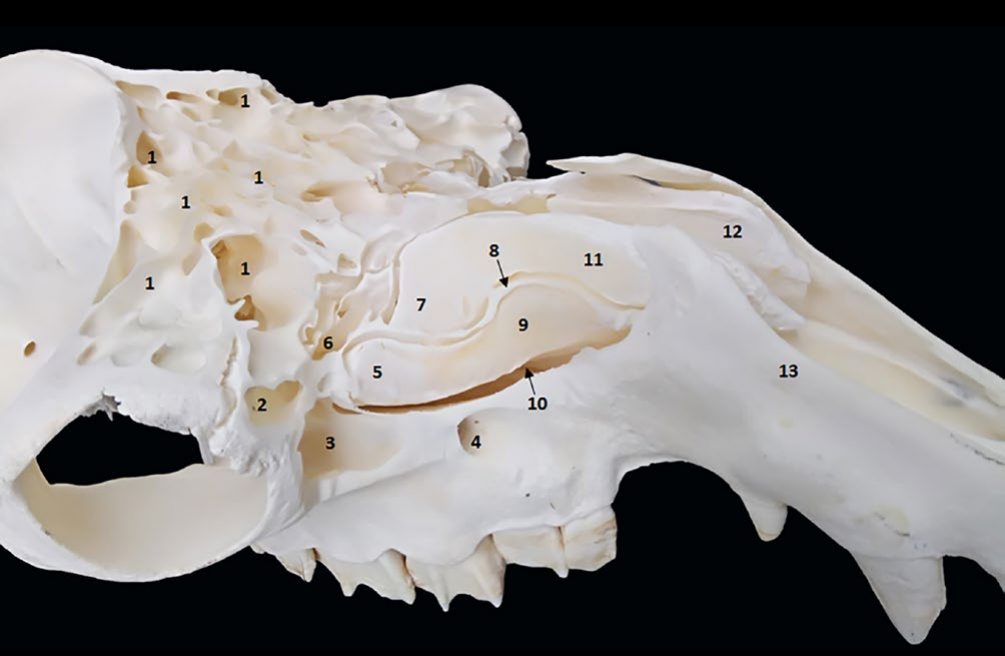
Oblique view of the paranasal sinus of dromedary. 1. Diverticula of frontal sinus; 2. lacrimal sinus; 3. maxillary sinus; 4. infraorbital foramen; 5. caudal part of the ventral nasal concha; 6. nasolacrimal duct; 7. rostral part of the dorsal nasal concha; 8. separation bone plate; 9. rostral part of the ventral nasal concha; 10. middle nasal meatus; 11. caudal part of the dorsal nasal concha; 12. left dorsal nasal concha; 13. nasal bone

Two parts in the conchal sinus group were identified: the dorsal and the middle conchal sinus (Figure 1 [5./7./9./11.]). The conchal dorsal sinus occupies the dorsal nasal meatus, whereas the middle conchal sinus occupies the ventral meatus (Figure 1 [10.]).

**FIGURE 2:**
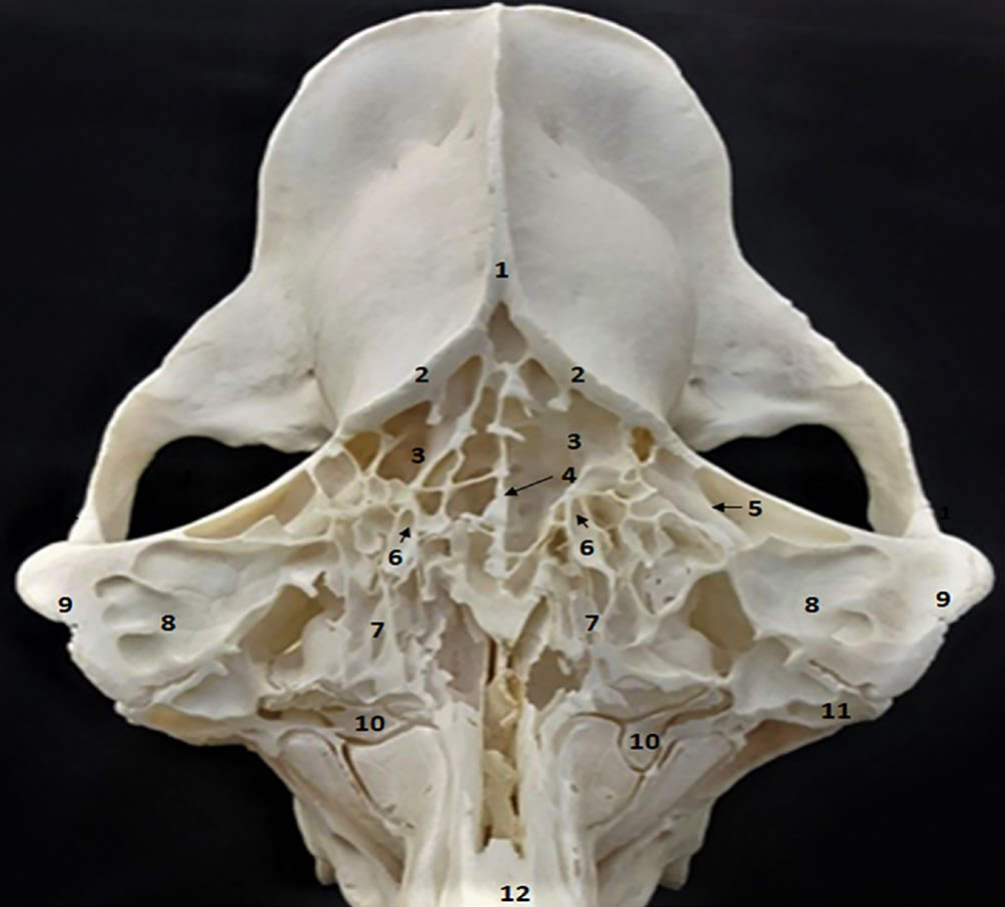
Dorsal view of the paranasal sinus of dromedary. 1. External sagittal crest; 2. temporal line; 3. caudal compartments of frontal sinus; 4. median interfrontal septum; 5. bony plates; 6. supraorbital foramen; 7. rostral compartments of frontal sinus; 8. lateral compartments of frontal sinus; 9. zygomatic process of frontal bone; 10. middle nasal concha; 11. lacrimal sinus; 12. nasal bone

The ventral conchal sinus is absent and replaced by spiral lamellae in the dorsal and ventral parts of the ventral nasal concha. The dorsal conchal sinus presents a pointed ventrolateral extension that partially extends towards the nasomaxillary opening (Figure 2 [10.]). The middle conchal sinus appears as a large ventral lateral extension to the caudal part of the ventral rostral nasal cornet from the screened plate and the dorsal to ethmoidal labyrinth up to the level of the third upper molar.

#### 3.1.2 Frontal sinus

**FIGURE 3:**
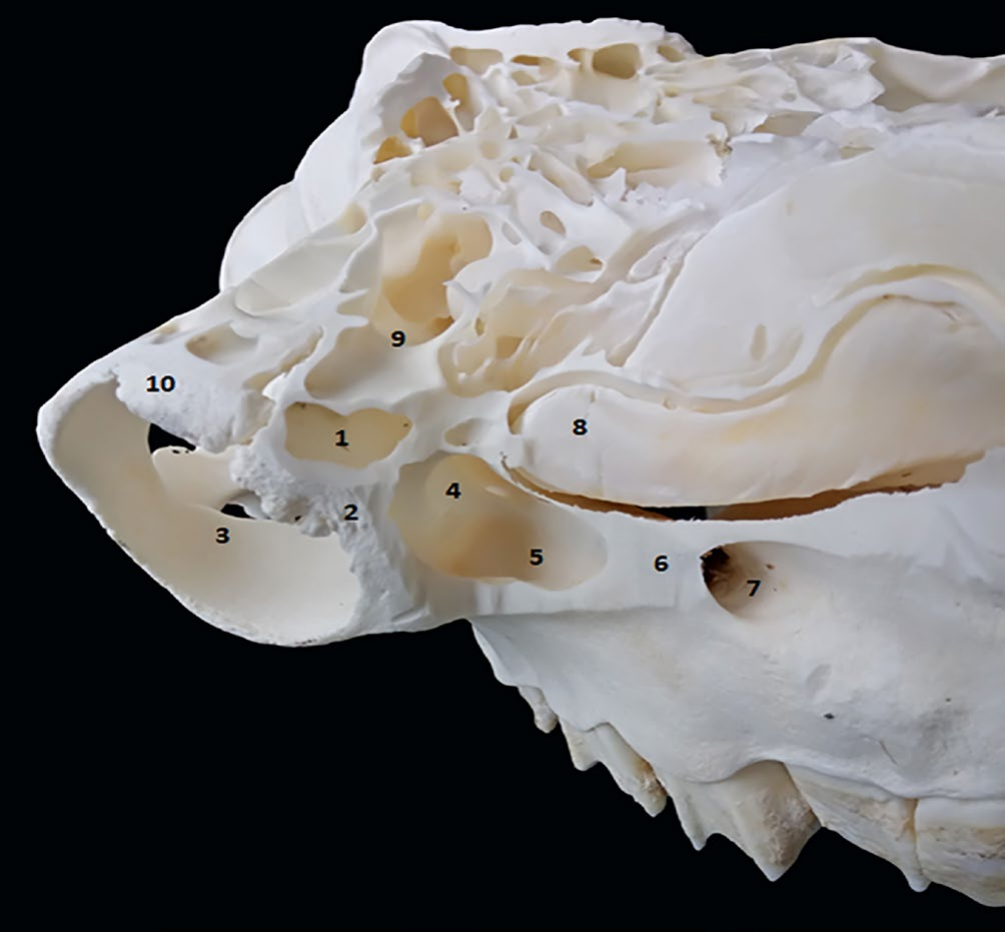
Close up‐ dorsal view of the paranasal sinus of dromedary. 1. Lacrimal sinus; 2. lacrimal bone; 3. orbit; 4. nasolacrimal duct; 5. maxillary sinus; 6. maxillary bone; 7. infraorbital foramen; 8. ventral nasal concha; 9. frontal sinus; 10. zygomatic process of frontal bone

The frontal sinus is very large in the frontal bone. It's divided into left and right sinuses by a complete medial bony interfrontum septum (Figure 3 [4.]). The right and left frontal sinuses are not symmetrical (Figure 3 [8.]). Indeed, the number of compartments in each sinus, the size and the separation are not similar in the two sinuses. Each frontal sinus is divided into cavities by numerous partitions of different sizes and positions between the different heads and between right and left frontal sinuses.

We identified large compartments delimited by thick and continuous partitions in each frontal sinus. These compartments are divided by smaller and discontinuous partitions into smaller compartments (Figure 1 [1.] and 3 [3./7.]). We noticed that the larger compartments were in the lateral position, whereas the cavities were smaller and have medial position. The supraorbital canal crosses the large caudolateral compartment (Figure 3 [6.]).

#### 3.1.3 Maxillary sinus

**FIGURE 4:**
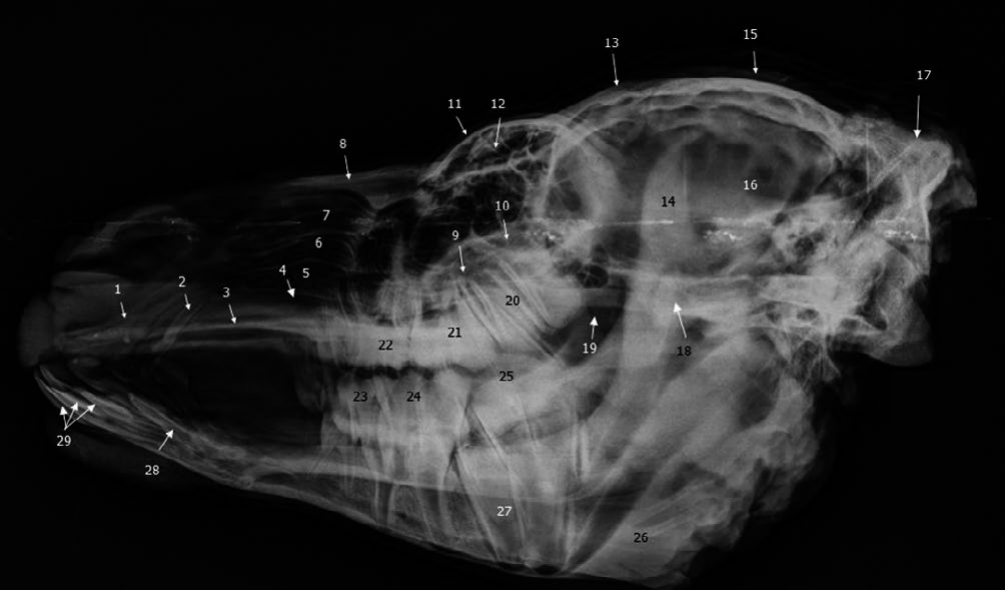
Lateral radiograph of dromedary head. 1. Incisive bone; 2. upper canine teeth; 3. vomer bone; 4. ventral nasal meatus; 5. ventral part of ventral nasal concha; 6. dorsal part of ventral nasal concha; 7. dorsal nasal concha; 8. nasal bone; 9. maxillary sinus; 10. lacrimal sinus; 11. orbit; 12. diverticula of frontal sinus; 13. frontal bone; 14. coronoid of mandible; 15. external sagittal crest; 16. cranial cavity; 17. parietal bone; 18. facial crest; 19. sphenoid sinus or oropharynx; 20. second upper molar; 21. first upper molar; 22. third upper premolar; 23. first lower molar; 24. second lower molar; 25. third lower molar; 26. ramus of mandible; 27. root of tooth; 28. lower canine teeth; 29. lower incisors

The maxillary sinus is a small sinus located in an excavation of a small part of the maxillary bone and the rostral part of the zygomatic bone (Figure 1 [3.] and 4 [5.]). The sinus cavity has triangular shape, its base is caudally directed and its cranial apex is located behind the infraorbital foramen. In dromedaries, the maxillary sinus extends to the level of the rostral edge of the third upper molar. This sinus communicates dorsally with the lacrimal sinus (Figure 4 [4.]) and the caudal part of the middle nasal meatus through the maxillolacrimal opening (Figure 2 [14.]). It extends to the level of the rostral edge of the third upper molar.

#### 3.1.4 Sphenoid sinus

**FIGURE 5:**
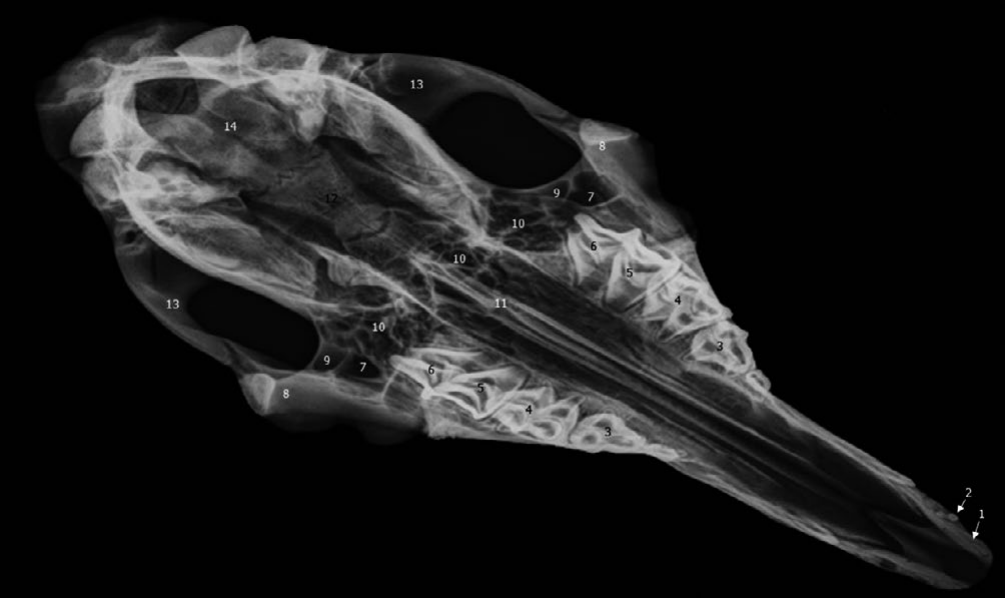
Dorsoventral radiograph of dromedary skull. 1. Incisive bone; 2. upper canine teeth; 3. third upper premolar; 4. first upper molar; 5. second upper molar; 6. third upper molar; 7. maxillary sinus; 8. zygomatic process of frontal bone; 9. lacrimal sinus; 10. diverticula of frontal sinus; 11. vomer bone; 12. basisphenoid bone; 13. zygomatic arch; 14. frontal bone; 15. occipital bone

The sphenoid sinus could only be identified in transverse and median sections, because it has a deep position under the skull: a cavity in the body of the sphenoid bone (Figure 5 [7.] and 6 [9.]). It's divided into small compartments by bony plates.

**FIGURE 6:**
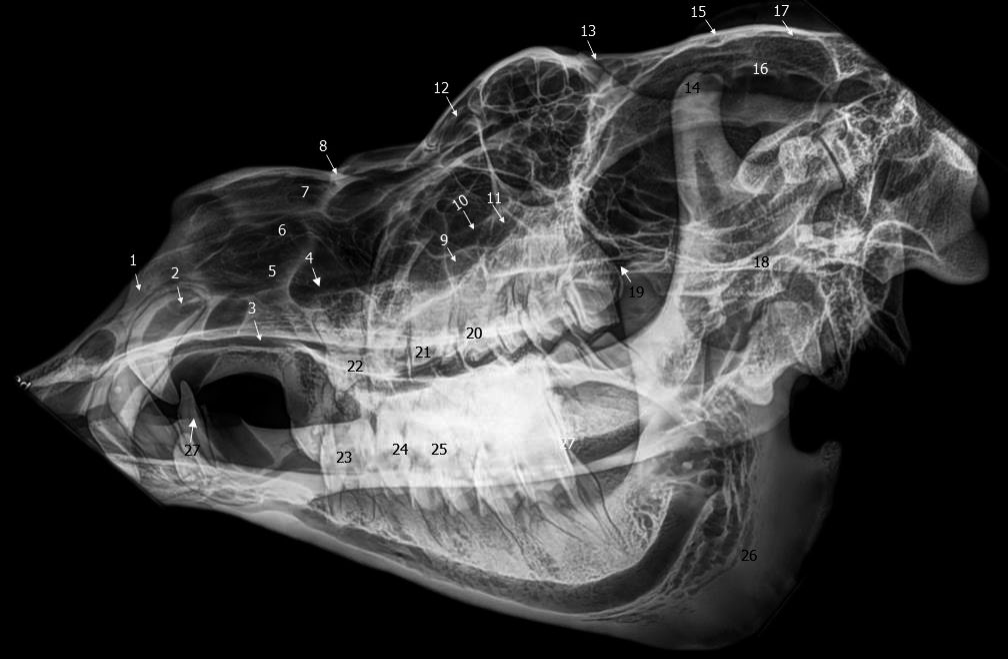
Oblique radiograph of dromedary skull. 1. Incisive bone; 2. upper canine teeth; 3. vomer bone; 4. ventral nasal meatus; 5. ventral part of ventral nasal concha; 6. dorsal part of ventral nasal concha; 7. dorsal nasal concha; 8. nasal bone; 9. maxillary sinus; 10. nasolacrimal duct; 11. lacrimal sinus; 12. diverticula of frontal sinus; 13. frontal bone; 14. coronoid of mandible; 15. external sagittal crest; 16. cranial cavity; 17. parietal bone; 18. facial crest; 19. oropharynx; 20. third upper premolar teeth; 21. second upper premolar teeth; 22. first upper premolar teeth; 23. first lower cheek teeth; 24. second lower cheek teeth; 25. third lower cheek teeth; 26. ramus of mandible; 27. lower canine teeth

#### 3.1.5 Ethmoid sinus

In the transverse sections, there are two cavities in the ethmoid bone located in the ethmoid labyrinths (Figure 5 [8.]). The ethmoid sinus communicates directly with the ethmoidal meatus and the nasal fundus. The ethmoid sinus of dromedaries consists of small cavities called ethmoid cells.

#### 3.1.6 Lacrimal sinus

The lacrimal sinus occupies a small cavity in the rostro‐medial lacrimal bone at the orbit (Figure 1 [2.], 2 [11.], 3 [11.] and 4 [1.]). Its lateral wall is constituted by the lacrimal bone, whereas the medial wall is constituted by the ethmoid bone. The lacrimal sinus is separated from the maxillary sinus by the nasolacrimal canal (Figure 2 [12.]) and communicates with the maxillary sinus by a maxillolacrimal opening (Figure 2 [14.]), anterior to the orbital cavity at the level of the third molar. The sinus cavity is not divided by bony plates and the nasolacrimal duct passes through its side wall.

In all dissected dromedaries' heads, we did not find a palatal sinus. In conclusion, the paranasal sinuses of the dromedary are represented only by frontal, maxillary, sphenoid, ethmoid and lacrimal sinuses.

### 3.2 Radiographic study of dromedaries' heads

**FIGURE 7:**
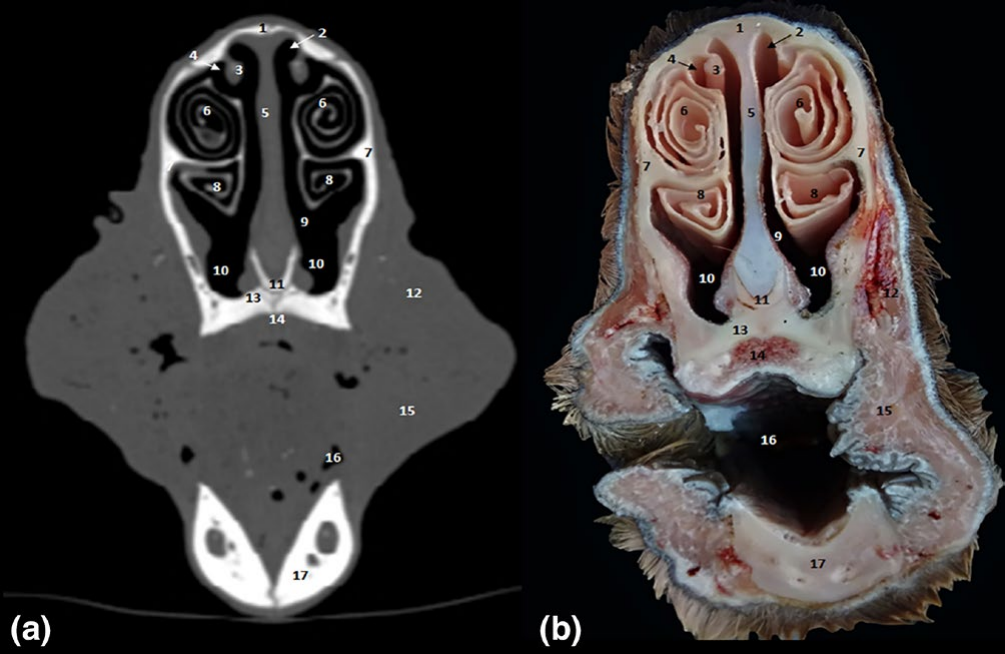
Cross‐sections at the level of the intermandibular joint. (a) Bone window CT image; (b) anatomic section. 1. Nasal bone; 2. dorsal nasal meatus; 3. dorsal nasal concha; 4. middle nasal meatus; 5. cartilage of the nasal septum; 6. dorsal part of ventral nasal concha; 7. maxillary bone; 8. ventral part of ventral nasal concha; 9. common nasal meatus; 10. ventral nasal meatus; 11. vomer; 12. levator nasolabialis muscle; 13. incisive bone; 14. hard palate; 15. buccinator muscle; 16. oral cavity; 17. intermandibular joint

In the present radiographic study, the lateral and the oblique views allowed exploring the frontal sinus with its compartments (Figure 7 [12.] and 8 [12.]), conchal sinuses with the nasal horns (Figure 7 [5./6.]), the maxillary sinus (Figure 7 [9.] and 8 [9.]) and the lacrimal sinus (Figure 7 [10.] and 8 [11.]). The limits of the sphenoid sinus and the ethmoid sinus are not identifiable due to the inevitable superposition of multiple opacities on these axially located structures. Sinus cavities are difficult to identify in dorsoventral views because the cheeks and overlying masseter muscles are superimposed on the field and only frontal and maxillary sinuses are visible (Figure 9 [7./10.]).

**FIGURE 8:**
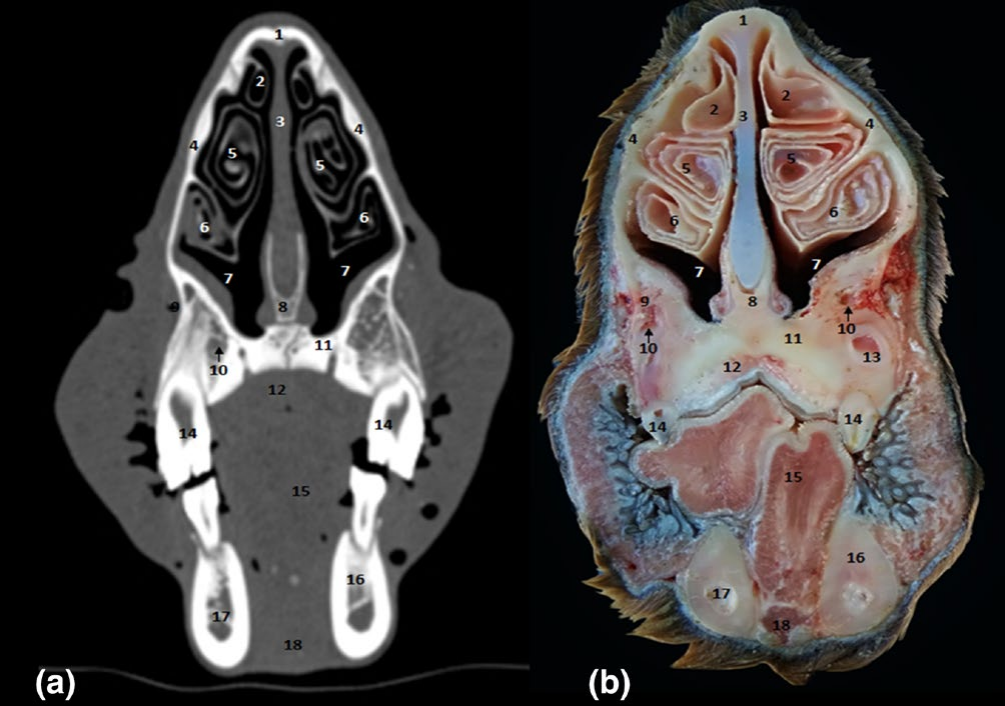
Cross‐sections at the level of second upper premolar. (a) Bone window CT image; (b) anatomic section. 1. Nasal bone; 2. dorsal nasal concha; 3. cartilage of the nasal septum; 4. axillary bone; 5. dorsal part of ventral nasal concha; 6. ventral part of ventral nasal concha; 7. ventral nasal meatus; 8. vomer bone; 9. maxillary sinus; 10. infraorbital canal; 11. incisive bone; 12. hard palate; 13. dental pulp; 14. second upper premolar; 15. tongue; 16. body of mandible; 17. mandibular canal; 18. geniohyoid muscle

**FIGURE 9:**
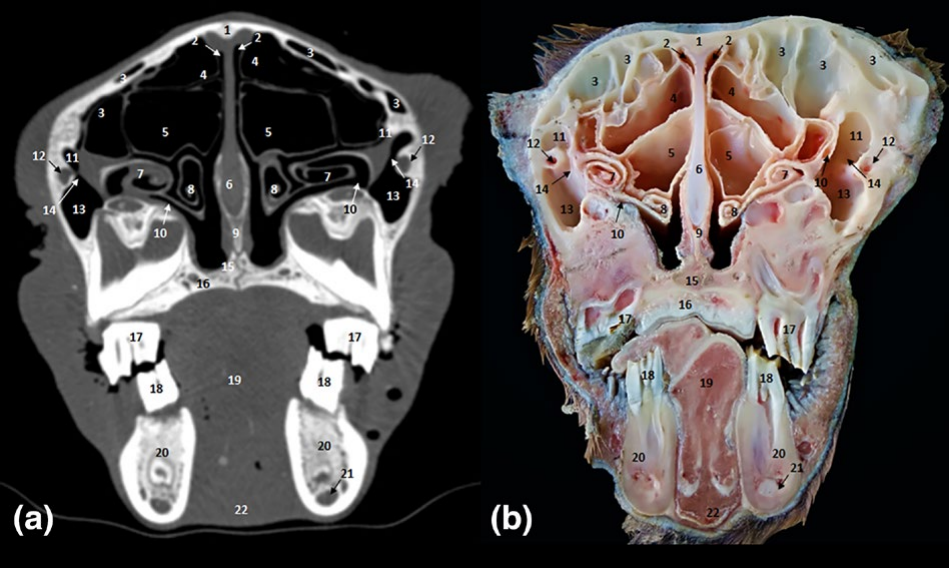
Cross‐sections at the level of the third upper premolar. (a) Bone window CT image; (b) anatomic section. 1. Frontal bone; 2. dorsal nasal meatus; 3. frontal sinus; 4. dorsal nasal concha; 5. middle nasal concha; 6. nasal septum; 7. dorsal part of ventral nasal concha; 8. ventral part of ventral nasal concha; 9. vomer bone; 10. nasomaxillary opening; 11. lacrimal sinus; 12. nasolacrimal canal; 13. maxillary sinus; 14., maxillolacrimal opening; 15. incisive bone; 16. hard palate; 17. third upper premolar; 18. third lower premolar; 19. tongue; 20. molar part of mandible; 21. mandibular canal; 22. genioglossus and geniohyoideus muscles

### 3.3 CT study

The CT study allowed performing 0.6‐mm‐spaced successive slices for the whole head. The paranasal sinuses topography and morphology of each sinus was studied. Sinuses were hypoattenuating air‐filled structures, bordered by thin, hyperattenuating bony walls.

**FIGURE 10:**
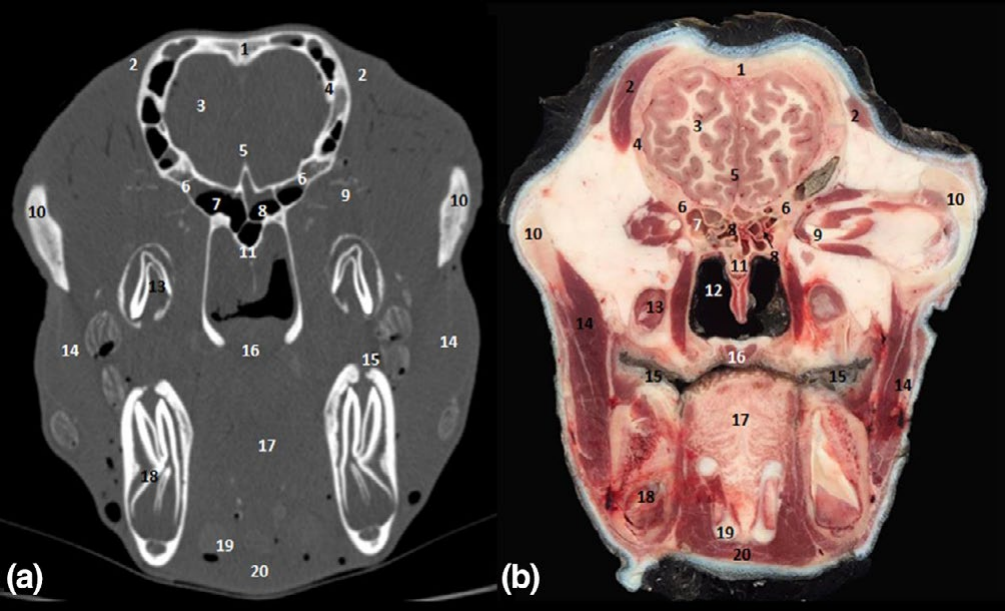
Cross‐sections at the level of the third lower molar. (a) Bone window CT image; (b) anatomic section. 1. Frontal bone; 2. temporal muscle; 3. frontal lobe of the brain; 4. parietal bone; 5. cerebral longitudinal fissure; 6. sphenoid bone; 7. sphenoid sinus; 8. endoturbinal volutes of ethmoid bone; 9. optic nerve; 10. zygomatic process of temporal bone; 11. vomer bone; 12. pars nasalis pharyngi; 13. root pulp; 14. masseter muscle; 15. palate diverticulum; 16. soft palate; 17. tongue; 18. lower third molar M3; 19. thyrohyoid bone; 20. geniohyoid muscle

CT scans allowed images of the fine bone and soft tissue architecture of both paranasal sinuses and nasal cavity. Paranasal sinuses of the dromedary consisted of the dorsal concha (Figure 10 [3.] and 11 [2.]), the middle concha (Figure 10 [4.] and 11 [5./6.]), maxilla (Figure 2 [13.]), frontal (Figure 2 [3.] and 6 [11.]), sphenoid (Figure 5 [7.] and 6 [9.]), lacrimal (Figure 2 [11.]) and ethmoid (Figure 5 [8.]) bones. These sinuses were identified and labelled according to the cheek teeth as landmarks. Communications between the paranasal sinuses were detectable by CT and macroscopic anatomy (Figure 2 [14.]).

**FIGURE 11:**
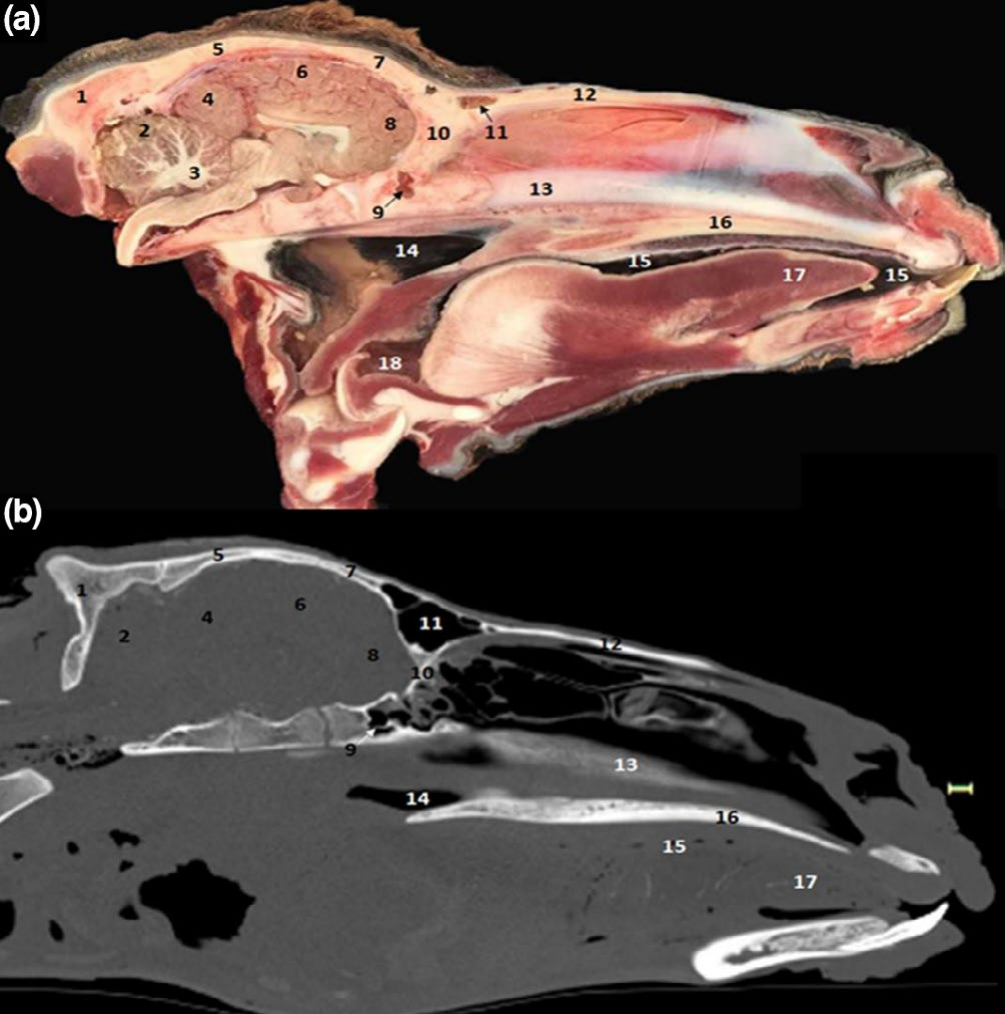
Sagittal sections of the head. (a) anatomic section; (b) Bone window CT image. 1. Occipital bone; 2. cerebellum; 3. medullary body of the cerebellum; 4. occipital lobe of the brain; 5. parietal bone; 6. parietal lobe of the brain; 7. frontal bone; 8. frontal lobe of the brain; 9. sphenoid sinus; 10. ethmoid bone; 11. frontal sinus; 12. nasal bone; 13. nasal septum; 14. nasopharynx; 15. oral cavity; 16. hard palate; 17. lingual apex; 18. oropharynx

## 4 DISCUSSION

Few studies explored dromedary paranasal sinuses with advanced imaging technology. To our knowledge, this is the first study where anatomical sections, bone fenestration by friction, radiographs and CTs scans were used to explore dromedary's sinuses. High quality images were obtained with a good correspondence between the anatomical sections and CT images. Other studies on other anatomical regions, such as carpus (Badawy et al., 2016) and metatarsus and fingers (El‐Shafey & Kassab, 2013), showed the interest of CT in dromedaries.

Opening windows through bone friction is a technique that was used by Al Safy et al., (2013) for the description of the paranasal sinuses of the Egyptian buffalo (*Bubalus bubalis*). This meticulous technique allows visualising the exact extent of each sinus and their divisions as well as potential communications between them. The anatomy in section allows a correct morphological and topographical study, it constitutes a useful tool for the identification of structures in CT images (De Zani et al., 2010; Smallwood et al., 2002). In the present study, transverse 1.5‐cm‐spaced anatomical sections were made with a band saw. The head was well frozen but we were not able to get thinner slices because the used band did not allow it. A smaller thickness could allow visualising of more anatomical structures. In addition, the presence of paramedian and dorsal anatomical sections could provide better details about the topography of sinuses and their relative ratios.

We identified two parts in the conchal sinus group, the dorsal and the middle conchal sinus, but there is no ventral conchal sinus in dromedaries. Alsafy et al., (2013) examined buffalo's paranasal sinuses and infirmed the presence of conchal ventral sinuses in buffalos, dromedaries and equines.

We found that the middle conchal sinus appears as a large extension in dromedaries. Alsafy et al., (2014) reported that, in dromedaries, this part communicates with the nasal fundus through a large caudal opening in its roof. These authors reported also that there is no sinus in the ventral nasal concha and the nasal blade is divided only into two spirals: dorsal and ventral.

The cornets and the middle nasal sinuses are larger and spread more rostrally in dromedaries than in cattle and buffaloes. According to Andrew and Lawrence (2004), the middle and dorsal meatus are narrower and the ventral meatus in dromedaries is wider than in horses, which may also be explained by the adaptation of dromedary to arid climate. Dromedary nose cones have special properties that differ from those of horses, buffaloes and cattle minimising dust and sand inhalation (Alsafy et al., 2013; Morrow & Park, 2000).

In the frontal sinus, we identified large compartments delimited by thick and continuous partitions. Alsafy et al., (2014) counted these compartments and found that each frontal sinus is divided into six large compartments by bony plates (caudal, lateral and rostral, two of each, respectively) surrounding eight small compartments. We didn't observe this organised division in the dissected dromedary's heads. According to Blanco et al., (2015), each sinus consists of a labyrinth of smaller spaces communicating with the nasal floor through small openings that we identified in the dissected heads.

The supraorbital canal crosses the large caudolateral compartment. According to Ahmed et al., (1985), this supraorbital canal divides the frontal sinus into a bean‐shaped rostro‐lateral part and a smaller triangular‐shaped caudomedial part. Several authors consider the rostro‐lateral part of the frontal sinus as lacrimal and maxillary sinuses. Moustafa and Kamel (1964) considered it as a lacrimal sinus separated from the maxillary sinus by a common orifice.

According to Ahmed et al., (1985), the frontal sinus boundaries are limited to the frontal bone. However, we found that the excavation of the frontal sinus in the dromedary extends beyond the angle of divergence of the external parietal crest. These observations were also reported by Moustafa and Kamel (1964).

The frontal sinus appears smaller than that of other domestic ruminants, it is divided into large compartments surrounding small compartments (Blanco et al., 2015). This division is similar but different from that reported in cattle and buffaloes. Cattle and buffaloes have also a nuchal and horned diverticulum that extends into the frontal sinus (Alsafy et al., 2013).

We found the same topography of the maxillary sinus that was reported by several authors (Ahmed et al., 1985; Alsafy et al., 2013; Moustafa & Kamel, 1964; Saber, 1990). Alsafy et al., (2014) and Blanco et al., (2015) added that the medial limit of the lacrimal sinus is the nasolacrimal canal.

The sphenoid sinus is divided into small compartments by bony plates, and opens directly into the nasal bottom through the nasosphenoid opening (Alsafy et al., 2013; Blanco et al., 2015). These compartments are larger than those observed in other ruminants (Alsafy et al., 2014).

The ethmoid sinus was identified as two cavities in the ethmoid labyrinths as reported by others (Ahmed et al., 1985; Alsafy et al., 2014). Blanco et al.,(2015) added that the ethmoid sinus communicates also with either the frontal or the sphenoid sinus. The sinus of dromedaries consists of small cavities called ethmoid cells. Alsafy et al., (2014) found that these cells open in the ethmoidal meatus.

The lacrimal sinus occupies a small cavity in the rostro‐medial lacrimal bone of the orbit. This sinus is lacking in horses, and more extended in cattle than that of dromedaries, reaching the lacrimal and frontal bones covering the orbit (Farag et al., 2017).

In all dissected dromedaries' heads, there was no palatal sinus. The paranasal sinuses of the dromedary are represented by frontal, maxillary, sphenoid, ethmoid and lacrimal sinuses. They are different from those of cattle and buffaloes, in these species the palatine sinus is also absent (Alsafy et al., 2013). The dromedary's paranasal sinuses are small compared to the head size of this species but also to the sinuses of other animal species (Moustafa & Kamel, 1964). Indeed, Saber (1990) reported that the dromedary's nasal cavity is narrower and longer than that of ox, buffaloes and horses. In addition, the maxillary sinus contains neither rostral nor caudal parts, as described in horses (Morrow & Park, 2000). We didn't find any fronto‐maxillary opening, as present in horses (De Zani et al., 2010).

We noticed also the absence of sphenopalatine sinus in dromedaries, a characteristic of horses reported by several authors (Smallwood et al., 2002; Solano & Brawer, 2004). Dromedaries have a sphenoid sinus excavated in the body of the presphenoid bone. In addition, the middle nasal horn and its corresponding sinus are larger than in other ruminant species (Hathcock et al., 2003, 1995).

The parameters of the X‐ray beam were chosen in order to obtain a satisfactory visualisation of the fine bone structures. However, if an acquisition in “low dose” can allow a correct study of the ethmoid bone details where there is a strong natural contrast (air‐bone). This study is more difficult if this contrast disappears as it is the case when there is an ethmoidal filling (Ferri & Klossek, 2003, 1995). In the present radiographic study, we easily explored the frontal sinus with its compartments, conchal sinuses with the nasal horns, the maxillary sinus and the lacrimal sinus. However, the limits of the sphenoid sinus and the ethmoid sinus were not identifiable. Similar results were obtained by Tucker and Farrell (2001) who studied the sphenopalatine sinus of horses. They think that the presence of fluid or soft tissue provides a certain radio‐opacity to the sinus, potentially improving its visualisation.

In the present work, X‐ray does not allow a correct observation of the ethmoid and the sphenoid sinus. The extent of these sinuses is underestimated. Therefore, it is difficult to observe the extent of mucosal lesions, changes in the bone wall structure, fracture lines and bone displacement. Indeed, according to Okuyemi and Tsue (2002), radiography do not allows a good management of sinusitis. However, due to its affordability, radiography is often used in the evaluation of sinusal affections and both facial and mandibular trauma. Moreover, acceptable radiographs of the sinuses can be taken with a portable X‐ray tube device, because the facial bones are thin and air within the sinuses acts as a natural contrast agent for soft tissue and fluids.

CT provides more detailed information about the anatomy and abnormalities of paranasal sinuses than plain films. Compared with conventional radiographs, the major advantage of CT is lack of superposition and an enhanced demonstration of individual components of the skull. Moreover, the regions of interest have high inherent radiographic contrast and CT provides clear unobstructed images of teeth.

Sinuses are hypoattenuating air‐filled structures, bordered by thin, hyperattenuating bony walls. These sinuses are identified and labelled according to the cheek teeth as landmarks (Alsafy et al., 2014; Shojaei, 2008). CT provides more detailed information about the anatomy and abnormalities of paranasal sinuses than plain films. Indeed, CT scan provides higher definition of sinuses and is more sensitive than plain radiography for detecting sinus pathology, especially within sphenoid and ethmoid sinuses (Okuyemi & Tsue, 2002). CT is more sensitive in detecting affections by discerning normal and abnormal structures (Byers et al., 2007; Tietje et al., 1996).

The present work represents a useful support diagnostic tool for interpretation of tomographic images for veterinary practitioners. The multimodal approach offers a valid interpretation of the paranasal sinus region. Because only two cadaver heads were explored, further studies using the CT and exploring dromedary paranasal sinuses of both juvenile and adult animals is necessary to compare the normal relative positions and sizes of anatomic structures of dromedary's heads.

We explored in the present study heads of slaughtered animals. To minimise soft tissue post‐mortem changes, the CT study was performed within 2 hr after slaughtering. For that reason, several veins were air‐filled.

Several studies of head CT in llamas (Hathcock et al., 2003, 1995) and horses (Morrow & Park, 2000; Smallwood et al., 2002) applied similar CT parameters (kV, mA). However, in most cases, the slice thickness and image spacing were 5–10 mm, whereas we used 0.6 mm image spacing in the present study. We believe that a lower slice thickness increases resolution to identify different anatomical structures. Interestingly, the accuracy and clarity of the CT image depend on the spacing technique. Similarly, previous studies reported that a thinner thickness of slices increases the resolution of the CT image (Alshipli & Kabir, 2017; Katkar et al., 2016; Yao et al., 2016). Moreover, thinner slices increase image noise, the visibility of a small lesion could be improved by providing more diagnostic content (Nagel, 2007).

Unfortunately, the use of CT as a single diagnostic imaging tool is generally limited in veterinary practice, especially for large animals such as dromedaries (Alberto Arencibia et al., 2012; Arencibia et al., 2005; Nawal & Amr, 2018). In Tunisia, experimental work was carried out on cadaveric anatomical parts, but not on living animals. This difficulty is mainly due to the absence of veterinary medical imaging centres adapted to dromedaries.

## 5 CONCLUSION

The compilation of gross sectional anatomy and different imaging techniques allowed a better understanding of the normal anatomy of the paranasal sinus region of adult dromedaries. The relationship between these complex anatomical structures was easily visualised. The compilation of anatomical sections, radiographic images and CT images allowed us to deepen the knowledge of topography and communications on the normal structure of the sinuses for a better interpretation of medical imaging, making easy the diagnosis of several head conditions, mainly tumours and inflammatory diseases. The huge development of imaging exploration tools of the paranasal sinuses allows a better knowledge of the anatomy of this region. We suggest performing a CT scan study on a larger sample of animals to mainly assess variations according to the dromedary's breed and age.

## CONFLICT OF INTEREST

There is no conflict of interest.

## AUTHOR CONTRIBUTION

Abdelmonem Ben Khalifa: Conceptualization; Data curation; Investigation; Methodology; Resources; Supervision; Writing‐original draft; Writing‐review & editing. AYOUB BEN BRAIEK: Methodology. LEILA BELHAJ HMIDA1: Investigation; Methodology. WALID CHANDOUL: Funding acquisition; Methodology. ABDELHAMID MATTOUSSI: Supervision; Validation; Writing‐review & editing.

### PEER REVIEW

The peer review history for this article is available at https://publons.com/publon/10.1002/vms3.515.
